# Adaptation and Validation of the Brazilian Portuguese Version of the Mental Health Knowledge Schedule (MAKS-BR) in the Context of Primary Health Care

**DOI:** 10.3390/ijerph22121809

**Published:** 2025-11-30

**Authors:** Larissa Moraes Moro, Vinícius Perinetto Pontel, Clarissa Pinto Pizarro de Freitas, Adriane Xavier Arteche, Kátia Bones Rocha

**Affiliations:** Post-Graduate Program in Psychology, Departament of Psychology, School of Health and Life Sciences, Pontifical Catholic University of Rio Grande do Sul (PUCRS), Porto Alegre 90619-900, SP, Brazil; larissamoraesmoro@gmail.com (L.M.M.); viniciusperinettopontel@gmail.com (V.P.P.); clarissa.freitas@pucrs.br (C.P.P.d.F.); adriane.arteche@pucrs.br (A.X.A.)

**Keywords:** mental health, primary health care, stigma, adaptation, validation, psychometrics

## Abstract

This study aimed to translate the Mental Health Knowledge Schedule (MAKS) to Brazilian Portuguese, adapt it to the Brazilian primary health care context, and evaluate its psychometric properties. The adaptation process involved three stages: translation, back-translation, and peer-group evaluation. To achieve a valid and reliable instrument, the Brazilian Portuguese version of the questionnaire (MAKS-BR) was administered through an anonymous, online self-administered questionnaire to a convenience sample of 289 primary care professionals with a mean age of 39.6 years (SD = 9.6 years), 90% of whom were women. The instrument, in its final 11-item model, presented satisfactory fit and comparative indices with a two-factor model. The first factor aggregates items related to mental health knowledge, while the second included items related to recognition and familiarity with various mental health conditions. The network findings support the discriminant validity of the two dimensions, while also underscoring the integrative nature of mental health literacy, in which knowledge and recognition processes are interconnected yet functionally distinct. Our findings suggest that the MAKS is an adequate instrument for assessing mental health knowledge, considering the linguistic and cultural contexts of Brazil.

## 1. Introduction

Mental health stigma can be understood as an umbrella concept that encompasses three core domains: a problem of knowledge (lack of knowledge about mental illness processes/ignorance), a problem of attitudes (prejudice), and a problem of behavior (discrimination) [1,2]. The social process of stigmatization related to people with mental health problems is a significant barrier to effective access to and care in health [3]. Deinstitutionalization of mental health services and implementation of community-based care represented important advances; nevertheless, these changes have not resulted in significant reductions in the stigma associated with mental disorders, neither globally nor in Brazil [2,4].

According to the World Health Organization [5], community integration only reduces fear and judgment when accompanied by cultural transformations, guaranteed rights, and greater social participation. Without those, negative attitudes, stereotypes, and discrimination toward people with mental health problems continue to prevail, which aggravates health conditions and limits the full exercise of citizenship [6,7]. In this scenario, Primary Health Care (PHC) serves as a strategic agent for organizing health systems, as it is the main point of entry for users and holds significant potential to reduce the treatment gap and expand coverage for priority mental health conditions [5]. However, research indicates that PHC physicians and nurses often express stigmatizing attitudes, especially toward conditions such as schizophrenia. This translates into less treatment adherence, delays in specialized referrals, and restrictive clinical practices [8,9]. Studies also point to key factors related to stigma showing that lower levels of knowledge, older age, and lack of interpersonal contact are associated with higher levels of stigmatization [10,11,12].

Additionally, studies reinforce the direct relationship between knowledge, attitudes and behaviors related to stigma and mental health treatment and recovery processes. In PHC, low levels of knowledge among professionals, stigmatizing attitudes, and discriminatory behaviors are associated with social exclusion and lower rates of seeking health services, compromising access and quality of adequate care and treatment [10,11,13,14,15]. Interventions aimed at reducing stigma are effective when they provide information that contributes to the level of knowledge of PHC professionals [16,17]. This is of relevance given that the professional’s knowledge of mental health relates to early recognition, referral, and continuity of care [5].

Albeit largely observed in health services, mental health stigma is widespread and people who experience mental health difficulties often have their human rights violated in multiple contexts, including limited access to economic and social resources, difficulties in joining the labor market, educational barriers, as well as limitations on the exercise of reproductive rights and access to the highest level of health [5,18,19]. Stigma also sustains the spread of myths such as the allegedly inability to work and recover; and misconceptions about the causes of mental health problems, often attributed to factors such as genetic inheritance, childhood experiences, education, aging, and intellectual disability [20].

One of the key strategies for reducing stigma in mental health is promoting knowledge about mental health and direct contact with people with mental disorders [2]. Based on the relevance of knowledge as an element related to stigma, researchers in the United Kingdom developed the Mental Health Knowledge Schedule (MAKS)—a scale designed to measure mental health knowledge [21,22,23]. Due to its simple, straightforward, and easy to understand format, the MAKS has been translated, adapted, and applied in various countries and contexts [24,25,26,27,28,29,30,31,32,33,34,35]. Although widely applicable, the adaptation of MAKS may vary, reflecting the heterogeneity of the items and mental health knowledge it assesses [21].

Despite knowledge about mental health being a central factor that influences attitudes, behaviors, and practices related to mental health, few studies specifically evaluate it. This gap is also observed when the assessment of knowledge about mental health is considered. There is a relative diversity of instruments adapted and validated for the Brazilian context aimed at assessing stereotypes and stigmatizing attitudes towards people with mental health problems, however, none have been validated to measure mental health knowledge. Therefore, this study addresses the question: does the MAKS demonstrate adequate psychometric properties for use in assessing the mental health knowledge of primary health care professionals in Brazil? Given this, the aim of this study was to translate the MAKS to Brazilian Portuguese, adapt it to the Brazilian primary health care context, and evaluate its psychometric properties.

## 2. Materials and Methods

### 2.1. The MAKS

MAKS covers evidence-based areas of knowledge on stigma reduction and was based on systematic literature reviews and consultations with experts, including researchers in the field of stigma as well as users of mental health services. As per authors’ suggestion, the instrument should be applied along with other measures that assess attitudes and behaviours related to stigma. The combined use of these instruments supports the planning and development of interventions targeting stigma reduction in mental health [21,22,23].

MAKS is an instrument specifically designed to capture the multidimensionality of mental health knowledge across different domains. The MAKS consists of 12 items. Items 1 to 6 assess mental health knowledge related to stigma: seeking help, recognition, support, employment, treatment, and recovery. Items 7 to 12 refer to levels of recognition and familiarity with various mental health conditions and help to contextualize the responses to other items. The items are presented on a 5-point Likert scale, from 1 for “strongly disagree” to 5 for “strongly agree”. The option ‘I don’t know’ is coded as neutral (value 3). Items 6, 8, and 12 are scored inversely. The score is calculated by summing up the responses given to the items, ranging from 12 to 60 points, with higher total scores corresponding to greater mental health knowledge. Higher and lower scores of respondents’ mental health knowledge may be related to specific areas of mental health. Higher total scores correspond to greater mental health knowledge.

The original construction study demonstrated moderate overall internal consistency for the first six items (Cronbach’s alpha = 0.65) and a reliability of 0.71 using Lin’s agreement [21]. The practical implication of this methodological heterogeneity is that Cronbach’s alpha, which primarily assesses the internal consistency of a unidimensional scale, is naturally attenuated. As noted by the original authors [21], because the MAKS is an intentionally diverse measure of multiple, related, but distinct domains (i.e., multiple factors), the overall internal consistency value is expected to be lower than that of a scale measuring a single, homogenous construct. Therefore, the obtained alpha of 0.61 is deemed acceptable and consistent with the scale’s design as a comprehensive assessment of diverse mental health knowledge facets.

### 2.2. Translation

The main authors of the original instrument were contacted and granted authorization for the translation and adaptation for Brazil. The translation of MAKS followed the guidelines and steps required by the authors [21]. The process involved three stages: translation, back-translation, and group evaluation. In the first stage, the original English version of the instrument was translated into Brazilian Portuguese by two Brazilian researchers who worked independently and whose first language is Portuguese, and second language is English. In the next stage, the translated versions were combined, and this version was back translated into English by two independent Brazilian Portuguese native speakers fluent in English. The translated and retranslated versions were then discussed with the study team and compared with the original English version to resolve any discrepancies and propose any necessary revisions to ensure the semantic, idiomatic and conceptual equivalence of the translated version.

### 2.3. Content and Face Validation

The group evaluation stage, a focus group with 10 researchers and research assistants, was conducted to establish the content validity and face validity of the MAKS-BR. The expert panel for content validity consisted of 10 professionals, including eight public health researchers specializing in mental health, and two psychometricians, all with experience in the Brazilian health care context. The format of the instrument, all items and instructions were discussed. Decisions sought to ensure semantic, linguistic and cultural equivalence between the original and translated instruments. A preliminary version of the instrument was presented, and each item was discussed. Experts evaluated each item for semantic, idiomatic, and conceptual equivalence with the original MAKS. They confirmed that the translated items adequately captured the domains of mental health knowledge as defined by the original scale, ensuring no domain was under-represented in the Brazilian context. Face validity was assessed by 10 health care professionals. They were asked to provide feedback on relevance, clarity, comprehensibility, and ease of response. To evaluate the final version of the scale, the following questions were asked for each item: “Is the statement clear?”; “Could the statement be reworded for better understanding?”; “How easy/difficult is it to choose an answer option for the statement?”. Items 1, 2, 3, 5, 6, 7, 8, 9, 10, 11, and 12 were considered clear and understandable by the professionals. In item 4, the original statement “Psychotherapy (e.g., talking therapy or counselling) can be an effective treatment for people with mental health problems” was adapted to the translated version “Psychotherapy can be an effective treatment for people with mental health problems”. The English terms talking therapy and counselling were removed to facilitate understanding, as in cultural terms, the use of the term psychotherapy is easily understood and commonly used in Brazil. Overall, respondents rated the MAKS-BR as highly relevant to their work and professional development, confirming the measure is suitable for assessing mental health knowledge in a Brazilian PHC context. Table 1 presents the items from the original instrument and the final translated version.

### 2.4. Participants

The sample consisted of 289 primary health care professionals. The sample size was defined considering the recommendation that confirmatory factor analysis (CFA) requires at least 10 participants per item, with a minimum of 250 participants, to ensure the robustness, stability, and reliability of the results [36]. The average age was 39.6 years (SD = 9.6, range 23 to 64 years), with 90.3% (*n* = 261) women; 74.7% (*n* = 216) self-declared white; and 34.6% (*n* = 100) married. Most respondents had postgraduate degrees (54%; *n* = 156) and had worked between five and ten years in primary health care services (30.8%, *n* = 89). Regarding mental health issues, 22.8% (*n* = 66) had some type of training to work in this area, and most had weekly contact with people diagnosed with mental disorders (27.9%; *n* = 80). Table 2 presents the sociodemographic characteristics of the sample.

### 2.5. Instruments

In addition to the MAKS-BR, participants answered a sociodemographic questionnaire. The questionnaire investigated variables such as gender, age, length of employment, education, training in the health field, and frequency of contact with family members, friends, or colleagues diagnosed with mental disorders.

### 2.6. Data Collection Procedures

The MAKS-BR was administered through an anonymous, self-administered online questionnaire on the Qualtrics platform. Recruitment occurred via the research group’s and researchers’ social media accounts, and the survey link remained open for four months. Furthermore, primary care service coordinators were invited to participate in the survey and to send the survey link to their teams. To access the instrument, participants had to indicate that they were over 18 years of age and agree to the Informed Consent Form. The voluntary nature of participation and guarantees of anonymity and confidentiality were emphasized, in line with ethical principles for research with human participants. To maintain data quality and integrity, the following control measures were implemented: duplicate response filters were used, employing the platform’s standard settings to prevent questionnaire resubmission from the same device or browser; and integrity review, which involved manual analysis of responses to identify patterns of incomplete or inconsistent filling (e.g., straight-lining). In total, 351 participants initiated the questionnaire, resulting in a response rate of 82.3%, with 289 participants completing the MAKS-BR. Participants with missing data for the MAKS-BR items were excluded from the final sample.

### 2.7. Data Analyses Procedures

Initially, descriptive analyses were conducted, including frequency distributions, means, and standard deviations for each item of the MAKS-BR. Floor and ceiling effects, which represent the proportion of participants who obtained the lowest and highest scores, respectively, on each item of the scale, were examined. Floor and ceiling effects were defined for each item by calculating the percentage of responses assigned to the first and last points of the Likert scale (1 = Strongly agree/ceiling; 5 = Strongly disagree/floor). A cut-off of 20% was adopted, such that values above or below this percentage indicated the presence of a floor or ceiling effect [37]. A Confirmatory Factor Analysis (CFA) was then performed to evaluate the fit of the MAKS-BR to a two-factor first-order oblique model, consistent with previous studies of the scale [21,23,24,35]. The first factor reflects knowledge about mental health (items 1–6), and the second factor reflects recognition and familiarity with various mental health conditions (items 7–12). The Weighted Least Squares Mean and Variance (WLSMV) estimator, appropriate for ordinal items, was employed. Model fit was assessed using the Comparative Fit Index (CFI), Tucker–Lewis Index (TLI), Root Mean Square Error of Approximation (RMSEA), and Standardized Root Mean Square Residual (SRMR), adopting the following criteria: CFI and TLI ≥ 0.90; RMSEA ≤ 0.08; and SRMR ≤ 0.08 [36].

Network analyses were conducted to examine the structural associations among the items of the MAKS-BR and their relations with sociodemographic variables (age, sex, education, work experience, mental health education and contact frequency with a person diagnosed with mental disorder). A polychoric correlation matrix was estimated, given the ordinal nature of the items. The network structure was then modeled with the Extended Bayesian Information Criterion graphical least absolute shrinkage and selection operator (EBICglasso) estimator [38], which has been shown to provide stable and replicable network solutions for psychological data and allows the identification of communities of items. Stronger associations are represented by shorter distances and thicker edges. Positive associations were shown through blue edges, and negative through red edges.

Centrality analyses of strength (the sum of absolute edge weights directly connected to a node), betweenness (the extent to which a node lies on the shortest paths between other nodes) and closeness (the average distance of a node to all others) were performed to evaluate the relative importance of nodes in the network. In addition, expected influence (EI) was estimated, which accounts for the direction of associations and has been recommended as a more robust centrality index in psychopathology and mental health research [39]. Finally, Bridge Expected Influence (EI1 and EI2) was computed to identify nodes that act as bridges between theoretically distinct communities in the network [40]. Bridge Expected Influence assesses the extent to which a node from one community (e.g., mental health knowledge items) connects to nodes in another (e.g., recognition and familiarity with various mental health conditions items or sociodemographic variables). Both 1-step (direct connections) and 2-step (indirect connections) bridge indices were examined, providing a fine-grained view of the cross-community influences within the network. All analyses were conducted in R Studio [41], using the packages lavaan [42], qgraph [43], bootnet [44], and networktools [40].

## 3. Results

Descriptive analysis of the professionals’ responses showed that the highest rates of full agreement were observed in items associating specific clinical conditions with the mental health category: items 9 (‘Schizophrenia is a type of mental illness’) and 10 (‘Bipolar disorder is a type of mental illness’), which obtained 93.8% and 89.6% of ‘Strongly agree’ responses, respectively. On the other hand, items 5 (‘People with serious mental health problems can recover completely’) and 1 (‘Most people with mental health problems want to have a paid job’) showed the highest percentages of ‘Neither agree nor disagree/Don’t know’ responses (14.5% and 11.8%). In contrast, the highest rates of complete disagreement emerged in items 8 (‘Stress is a type of mental illness’) and 12 (‘Grief is a type of mental illness’), with 40.8% and 30.4% of ‘Strongly disagree’ responses, respectively (Table 3). The average score of participants in the mental health knowledge dimension was 12.6 (SD = 3.7), ranging from 6 to 36. The dimension of recognition and familiarity with various mental health conditions obtained an average value of 10.6 (SD = 4.3), with values ranging from 6 to 36.

A significant ceiling effect was observed in 9 of the 12 items (Items 1, 2, 3, 4, 7, 9, 10, 11, and 12). This is because a large proportion of primary health care professionals selected the “Strongly Agree” option. A significant floor effect was observed in 2 of the 12 items (Items 8 and 12). The high concentration of “Strongly Agree” responses (especially in items 9 and 10) suggests that most participants have a good level of knowledge about these topics.

The results of the CFA for the two-factor oblique first-order model, comprising Mental Health Knowledge (MHK, items 1 to 6) and Recognition and Familiarity with Various Mental Health Conditions (RFMH, items 7 to 12), demonstrated satisfactory psychometric properties (χ^2^(53) = 148.90, *p* < 0.001, CFI = 0.947, TLI = 0.934, RMSEA = 0.079 (IC90% = 0.064–0.094), SRMR = 0.084). The association between the MHK and RFMH was 0.58 (*p* < 0.001), suggesting a moderate association. This pattern indicates that the factors share substantial variance while maintaining conceptual distinctiveness. Standardized factor loadings ranged from 0.324 to 0.650 for MHK and from 0.534 to 0.832 for RFMH. All items except item 6 loaded significantly on their respective factors, indicating that it does not meaningfully contribute to the latent factor (Table 4). The threshold analysis of the MHK dimension indicated that items 5 and 1 were the easiest to endorse, whereas items 3 and 4 required greater levels of the latent trait for endorsement (Table 4). In MHK, item 6 stood out negatively, as it failed to load significantly on the factor and displayed unstable thresholds, thus emerging as the most problematic item within this dimension. In the RFMH dimension, items 11 and 12 were identified as the easiest, while items 9 and 10 were found to be the most difficult within this factor (Table 4). Furthermore, MHK showed low internal consistency (ordinal α = 0.59, ω = 0.45).

Considering that item 6 did not present a significant factor loading in the MAKS first model assessed, a second CFA removed item 6 and maintained the two-factor oblique first-order model. The revised 11-item solution showed satisfactory fit indices (χ^2^(43) = 126.95, *p* < 0.001, CFI = 0.953, TLI = 0.940, RMSEA = 0.082 (90% CI 0.066–0.099), SRMR = 0.085). The MHK and RFMH relation remained moderate (r = 0.58, *p* < 0.001). Items standardized factor loadings were adequate for both dimensions (MHK = 0.325 to 0.650, RFMH = 0.534 to 0.832. The item-threshold ordering was similar to the first CFA evaluated, within MHK, items 5 and 1 remained easiest and items 3 and 4 most demanding; within RFMH, items 11 and 12 were easiest and items 9 and 10 the most difficult (Table 4). Eliminating item 6 produced a more coherent MHK factor, improving its internal consistency indices (ordinal α = 0.66, ω = 0.53).

The network analysis revealed that the dimensions of MHK and RFMH formed coherent and clearly distinguishable communities, consistent with theoretical expectations. The community detection procedure indicated the presence of relatively independent modules, which nonetheless remained interconnected through meaningful cross-links, suggesting that while the constructs capture distinct aspects of mental health literacy, they are not entirely isolated domains. Within the MHK dimension, the most pronounced associations were found among items 1, 3, 4, and 5, suggesting a tightly connected subnetwork reflecting core knowledge elements. Conversely, in the RFMH dimension, the strongest edges were identified among items 7, 9, 10, and 11, highlighting a robust clustering of indicators related to the recognition of specific mental disorders.

**Figure 1:**
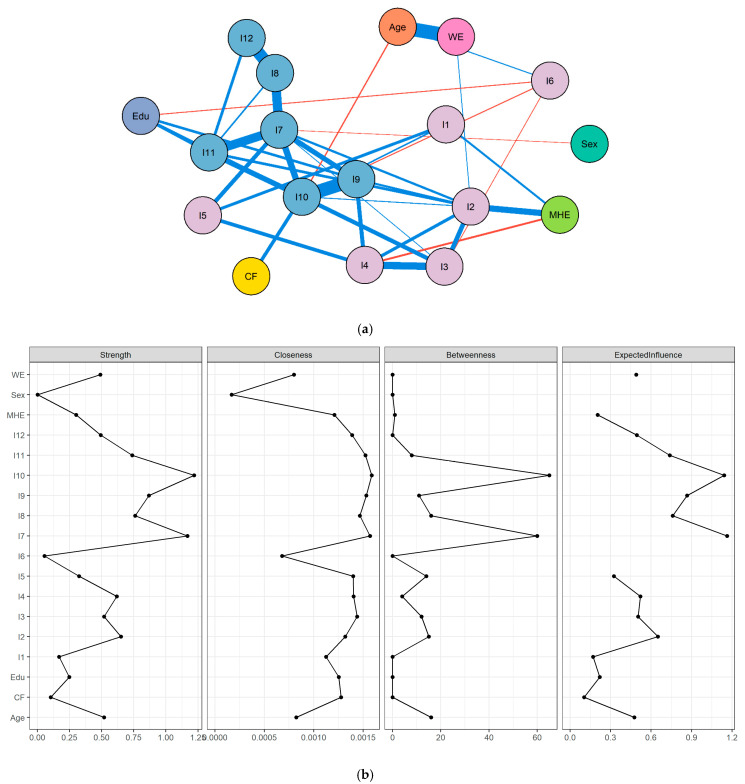

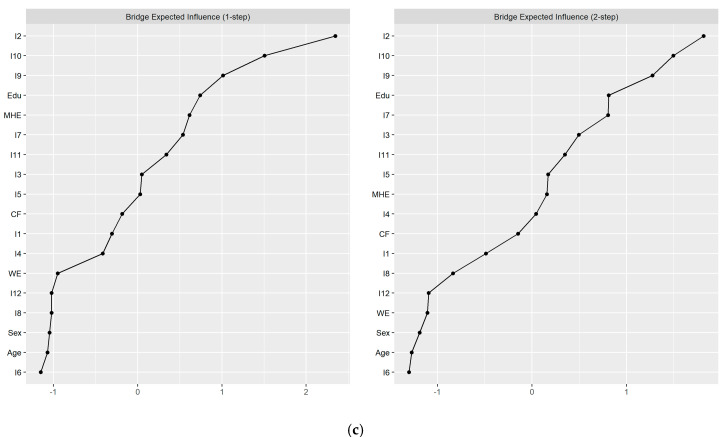
Network Analysis, Centrality Indices and Bridge Expected Influence of Mental Health Knowledge (MHK) and Recognition and Familiarity with Various Mental Health Conditions (RFMH) Items with Sociodemographic Variables. (**a**). Network analysis of MHK and RFMH items with sociodemographic variables; (**b**). Centrality indices (strength, betweenness, closeness and expected influence) for the network nodes; (**c**). Bridge expected influence (1-step and 2-step). Note: I1–I6 = Items Assessing Mental Health Knowledge, I7–I12 = Recognition and Familiarity with Various Mental Health Conditions, Edu = Education, MHE = Mental Health Education, CF = Contact Frequency with a Person Diagnosed with Mental Disorder, WE = Work Experience. Blue lines represent positive relations. Red lines represent negative relations.

With respect to sociodemographic variables, higher levels of education were associated with greater ability to accurately identify mental disorders, particularly items 7 and 10. Likewise, the frequency of contact with individuals experiencing mental disorders was positively associated with greater knowledge of mental health (item 2) as well as with improved recognition of specific disorders (item 11). In contrast, sex, age, and years of professional experience were positioned at the periphery of the network, showing only weak associations and failing to exert a central structural role in the system. These findings suggest that educational attainment, professional training, and experiential contact function as key drivers of mental health literacy, whereas demographic factors such as gender and age appear to play only marginal roles in shaping these competencies (Figure 1a).

The centrality analysis evidence that RFMH items 7 and 10 exhibited the highest values across strength, betweenness, closeness and expected influence. Within MHK items 2, 3 and 4 emerged as key intermediaries, displaying consistent values of strength and betweenness (Figure 1b and Table 5). The results obtained through the Bridge Expected Influence (1-step and 2-step) analysis demonstrated that age and work experience acted as bridging variables linking otherwise distinct communities within the network (Figure 1c). Although these sociodemographic variables were not located at the densest core of associations (Figure 1a), they nonetheless conditioned the connections between the knowledge domain (items 1 and 6) and the disorder recognition domain (items 7 and 12) (Figure 1c and Table 5).

## 4. Discussion

This study aimed to translate and provide a description of psychometric properties of the MAKS-BR within a sample of primary health care professionals. MAKS-BR proved to be adequate for measuring the mental health knowledge of PHC professionals in Brazil. The instrument presents satisfactory fit and comparative indices with a two-factor model. The first factor aggregates items related to mental health knowledge (MHK), while the second includes items related to recognition and familiarity with various mental health conditions (RFMH). The network findings support the discriminant validity of the two dimensions, while also underscoring the integrative nature of mental health literacy, in which knowledge and recognition processes are interconnected yet functionally distinct.

Regarding psychometric results, the MHK factor initially presented a Cronbach coefficient of 0.59. Low or moderate values of internal consistency are expected in this context, considering that the MAKS was developed to assess a heterogeneous set of items, in which respondents’ knowledge may manifest itself selectively in different areas of mental health [21]. Of the 12 items in the instrument, only item 6 presented problems of psychometric adequacy, which was also identified in the French adaptation, in which its factor loading was not significant [27]. Beyond these psychometric issues, item 6 was removed on substantive grounds: its wording was overly broad, which hindered comprehension and compromised interpretability of the underlying construct. Consistent with prior evidence, excluding item 6 in the present validation improved internal consistency indices for the MHK domain, mirroring the French findings [27]. It is worth noting that the very design of the MAKS does not expect high internal consistency. Thus, although the removal of item 6 is psychometrically adequate and replicated in different contexts, the reliability indices should be interpreted in light of this characteristic of the instrument, which favors the diversity of content evaluated over internal consistency [21]. To retain the conceptual coverage of item 6 regarding help-seeking while reducing its ambiguity, future studies should investigate the contribution of the item reformulated to: “People who experience mental health problems are likely to seek help from a qualified healthcare professional (e.g., psychiatrist, psychologist, or primary care physician) when they face significant distress or functional impairment.”. This revision narrows the referent (“qualified professional”) and specifies the condition for help-seeking (“significant distress/impairment”), thereby enhancing interpretability and expected psychometric performance.

Another relevant aspect of the psychometric analysis refers to the ordering of items by levels of difficulty, which reinforces the sensitivity of the MAKS-BR in capturing variations in different degrees of mental health knowledge. This pattern suggests that the instrument not only assesses the construct in global terms, but also discriminates between different levels of proficiency, which contributes to its validity. The presence of items ranging from more basic content to more complex conceptual demands increase the accuracy of the measurement, ensuring greater robustness in identifying gradients of mental health literacy among PHC professionals.

When analyzing the relationships between the items in the instrument and the sociodemographic variables, it is observed that professionals who have more contact with people with mental health problems have greater skills in identifying mental disorders and greater knowledge of mental health. This suggests that direct contact with individuals with mental health problems can function as a practical learning mechanism, allowing PHC professionals to integrate concrete experiences into their theoretical and clinical repertoire. This result is consistent with an umbrella review of 216 review articles, which showed that forms of social contact, direct or indirect, between people who have and who do not have lived experience in mental health constitute the most effective, evidence-based strategy for reducing stigma and increasing knowledge [2].

However, previous studies indicate that it is not the frequency of contact that predicts better levels of mental health literacy or stigma reduction, but rather the quality of interactions [45,46]. Evidence indicates that stigma related to mental disorders is not associated with the amount of contact and that, in certain contexts, prolonged exposure of health professionals can exacerbate stigmatizing attitudes due to occupational stress or negative experiences of interaction [47,48]. Thus, an increase in positive interactions seems to contribute to mitigating the stigma associated with people with mental health problems [49].

Another important finding of this study is that professionals with higher levels of education showed greater ability to recognize mental disorders, a result consistent with previous studies [50,51]. Considering that PHC is the gateway to health services and that access to mental health care is still limited, it is essential that all staff, including physicians, nurses, nursing technicians, and community health workers, have a basic level of knowledge about mental health. This finding highlights the importance of continuing education in health, especially for professionals with lower educational levels, since ongoing training strategies can strengthen knowledge, reduce stigma, and improve the quality of care and management of users with mental health problems in PHC [5].

Instruments that assess aspects related to stigma play a fundamental role in different healthcare settings in Brazil. In this sense, MAKS-BR can be used as a strategic tool by professionals and researchers to assess teams’ mental health knowledge, supporting the development of interventions aimed at expanding knowledge and reducing stigma.

From an applied perspective, the finding that items 7 and 10 function as highly central hubs suggests that the ability to recognize specific mental disorders may play an influential role in shaping overall mental health literacy. Interventions aimed at strengthening recognition skills for these disorders may therefore produce cascading benefits throughout the network, enhancing both general knowledge and recognition of other conditions. Similarly, the intermediary position of items 2, 3, and 4 within the MHK dimension indicates that educational initiatives discussing this thematics may contribute to broaden metal health literacy strategies. The results of the Bridge Expected Influence analysis suggest that younger professionals may possess greater skills in identifying mental disorders and, consequently, exhibit higher levels of RFMH. Conversely, greater work experience appears to support higher levels of MHK. Taken together, age and work experience may therefore play complementary roles in shaping mental health literacy.

This study used a sample of primary health professionals with an online data collection and an uneven sex distribution. Participants were recruited online and therefore a bias to engage in the topic may have operated. Future studies shall overcome these limitations using various recruitment strategies and stratified sampling (e.g., by region and professional group) to enhance representativeness and permit subgroup invariance testing.

## 5. Conclusions

MAKS-BR has proven to be a psychometrically adequate instrument for assessing mental health knowledge, considering the linguistic and cultural contexts of Brazil. In the context of PHC, mental health knowledge was influenced by multiple factors, including age, education, length of employment, and frequency of contact with people diagnosed with mental disorders. The validation of the MAKS-BR is significant because it is one of the first mental health knowledge scales adapted and validated specifically for primary health care providers in Brazil, filling a critical gap in assessing the foundational literacy required for integrated care. Furthermore, this study adopted a comprehensive psychometric approach by combining Confirmatory Factor Analysis (CFA) with network analysis. In addition to measuring knowledge, MAKS-BR can be used as a rapid tool to identify knowledge gaps before initiating a continuing education program. Additionally, it can be used in a pre and post-intervention design to evaluate the effectiveness of mental health training modules implemented at PHC.

Despite these strengths, the study has a few limitations that warrant consideration. The primary limitation is the use of convenience sampling, which limits the generalizability of the findings. The use of a convenience sample may have introduced a selection bias, resulting in a sample of PHC professionals potentially more engaged in mental health issues, which could explain the high knowledge scores observed. As the sample was drawn from a specific regional context, the results may not be fully representative of all primary health care professionals across the diverse linguistic and cultural regions of Brazil. Future research should therefore prioritize probabilistic sampling strategies and replicate the factor and network structures in other Brazilian states. Also, future research may use MAKS-BR in conjunction with other instruments that assess stigma, allowing for a deeper understanding of the relationship between knowledge, attitudes, and stigmatization in the context of PHC.

## Figures and Tables

**Table 1:** Original version and Brazilian version of the Mental Health Knowledge Instrument—MAKS-BR.

Item	Original Instrument	Brazilian Portuguese Translated and Adaptaded
1	Most people with mental health problems want to have paid employment	A maioria das pessoas com problemas de saúde mental quer ter um trabalho remunerado.
2	If a friend had a mental health problem, I know what advice to give them to get professional help.	Se um amigo tiver um problema de saúde mental, eu sei como aconselhá-lo para obter ajuda profissional.
3	Medication can be an effective treatment for people with mental health problems.	A medicação pode ser um tratamento eficaz para pessoas com problemas de saúde mental.
4	Psychotherapy (e.g. talking therapy or counselling) can be an effective treatment for people with mental health problems.	A psicoterapia pode ser um tratamento eficaz para pessoas com problemas de saúde mental.
5	People with severe mental health problems can fully recover.	Pessoas com sérios problemas de saúde mental podem se recuperar totalmente.
6	Most people with mental health problems go to a healthcare professional to get help.	A maioria das pessoas com problemas de saúde mental recorre a um profissional de saúde para obter ajuda.
7	Depression is a type of mental illness	Depressão é um tipo de doença mental
8	Stress is a type of mental illness	Estresse é um tipo de doença mental
9	Schizophrenia is a type of mental illness	Esquizofrenia é um tipo de doença mental
10	Bipolar disorder (manic-depression) is a type of mental illness	Transtorno bipolar é um tipo de doença mental
11	Drug addiction is a type of mental illness	Dependência de drogas é um tipo de doença mental
12	Grief is a type of mental illness	Luto é um tipo de doença mental

**Table 2:** Description of the sample’s sociodemographic data.

Variable	Characteristics	% (*n*)
Age	19–29	16.6 (48)
	30–39	37.7 (109)
	40–49	27.7 (80)
	50–59	15.9 (46)
	>60	1.7 (5)
Sex	Female	90.3 (261)
	Male	9.7 (28)
Race/color	White	74.7 (216)
	Brown	14.5 (42)
	Asian	1.0 (3)
	Black	8.0 (23)
	Indigenous	0.3 (1)
	Other	1.4 (4)
Marital status	Single	31.5 (91)
	Civil partnership	22.8 (66)
	Married	34.6 (100)
	Separated/divorced	10 (29)
	Widower/widow	0.7 (2)
Education	Primary education to incomplete secondary education	2.4 (7)
	Complete secondary education	18.3 (53)
	Incomplete higher education	9.3 (27)
	Comple higher education	15.9 (46)
	Postgraduate studies	54.0 (156)
Occupation	Nurse	31.5 (91)
	Community health worker	22.5 (65)
	Physician	12.5 (36)
	Nursing assistant or technician	9.3 (27)
	Dentist	6.9 (20)
	Psychologist	4.5 (13)
	Manager	4.2(12)
	Other	8.3 (24)
Working time	Less than 1 year	12.8 (37)
	1 to 3 years	15.9 (46)
	3 to 5 years	9.3 (27)
	5 to 10 years	30.8 (89)
	10 to15 years	12.5 (36)
	15 to 20 years	9.7 (28)
	More than 20 years	8 (23)
Mental health training	Sim	22.8 (66)
	Não	77.2 (233)
Frequency of contact with people with mental disorders	Daily	24.7 (71)
	Weekly	27.9 (80)
	Monthly	17.4 (50)
	Rarely	20.9 (60)
	Never	1.7 (5)

**Table 3:** Frequency and percentage of each response/item in the questionnaire.

Item	Agree Strongly		Agree Slightly		Neither Agree nor Disagree/Don’t Know		Disagree Slightly		Disagree Strongly	
	%	*n*	%	*n*	%	*n*	%	*n*	%	*n*
1. Most people with mental health problems want to have paid employment.	30.8	89	45.3	131	11.8	34	9.7	28	2.1	6
2. If a friend had a mental health problem, I know what advice to give them to get professional help.	58.5	169	35.3	102	4.5	13	1.0	3	0.7	2
3. Medication can be an effective treatment for people with mental health problems.	37.0	107	56.4	163	2.4	7	3.1	9	1.0	3
4. Psychotherapy can be an effective treatment for people with mental health problems	67.5	195	28.4	82	2.8	8	1.0	3	0.3	1
5. People with severe mental health problems can fully recover.	17.0	49	53.3	153	14.5	42	11.4	33	3.8	11
6. Most people with mental health problems go to a healthcare professional to get help.	12.1	35	35.6	103	7.6	22	33.9	98	10.7	31
7. Depression is a type of mental illness.	78.5	227	15.6	45	3.5	10	0.3	1	2.1	6
8. Stress is a type of mental illness.	8.0	23	6.6	19	8.7	25	36.0	104	40.8	118
9. Schizophrenia is a type of mental illness.	93.8	271	4.2	12	1.4	4	0.7	2	0	0
10. Bipolar disorder (manic-depression) is a type of mental illness.	89.6	259	8.7	25	1.4	4	0.3	1	0	0
11. Drug addiction is a type of mental illness.	60.2	174	26.0	75	4.2	12	6.6	19	3.1	9
12. Grief is a type of mental illness.	21.1	61	28.4	82	8.0	23	12.1	35	30.4	88

**Table 4:** Standardized Factor Loadings and Thresholds of MAKS-12 and MAKS-11.

MAKS-12		F.L.	S.E.	95% I.C.		Thresholds				
Factor	Item			L.	H.	*t*1	*t*2	*t*3	*t*4	*t*5
MHK	1	0.324 *		0.204	0.445	−0.502	0.722	0.970	1.482	1.660
MHK	2	0.645 *	0.566	0.524	0.765	0.214	1.536	1.817	1.973	2.113
MHK	3	0.650 *	0.430	0.555	0.745	−0.331	1.508	1.627	2.038	2.312
MHK	4	0.628 *	0.606	0.513	0.743	0.453	1.733	1.817	1.973	2.038
MHK	5	0.464 *	0.398	0.354	0.574	−0.956	0.531	0.791	1.280	1.536
MHK	6	−0.001	0.209	−0.138	0.135	−1.241	−0.135	−0.013	1.042	1.916
RFMH	7	0.819 *		0.729	0.909	0.791	1.565	1.864	1.916	2.461
RFMH	8	0.706 *	0.047	0.634	0.778	−0.232	0.744	0.916	1.187	1.774
RFMH	9	0.825 *	0.055	0.719	0.930	1.536	2.038	2.113	2.312	NA
RFMH	10	0.832 *	0.051	0.732	0.933	1.260	2.113	2.202	NA	NA
RFMH	11	0.709 *	0.043	0.621	0.796	0.259	1.087	1.187	1.627	2.038
RFMH	12	0.534 *	0.046	0.448	0.619	−0.803	−0.013	0.100	0.415	1.817
MHK	1	0.325 *		0.204	0.445	−0.502	0.722	0.970	1.482	1.660
MHK	2	0.645 *	0.439	0.524	0.765	0.214	1.536	1.817	1.973	2.113
MHK	3	0.650 *	0.411	0.555	0.745	−0.331	1.508	1.627	2.038	2.312
MHK	4	0.628 *	0.384	0.513	0.743	0.453	1.733	1.817	1.973	2.038
MHK	5	0.464 *	0.297	0.354	0.574	−0.956	0.531	0.791	1.280	1.536
RFMH	7	0.819 *		0.729	0.909	0.791	1.565	1.864	1.916	2.461
RFMH	8	0.706 *	0.068	0.634	0.778	−0.232	0.744	0.916	1.187	1.774
RFMH	9	0.825 *	0.078	0.719	0.930	1.536	2.038	2.113	2.312	NA
RFMH	10	0.832 *	0.068	0.732	0.933	1.260	2.113	2.202	NA	NA
RFMH	11	0.709 *	0.068	0.621	0.796	0.259	1.087	1.187	1.627	2.038
RFMH	12	0.534 *	0.058	0.448	0.619	−0.803	−0.013	0.100	0.415	1.817

Note: * = *p* < 0.001, MHK = Mental Health Knowledge, RFMH = Recognition and Familiarity with Various Mental Health Conditions, F.L. = Factor Loading; S.E. = Standard Error.

**Table 5:** Centrality Indices and Bridge Expected Influence Values of Mental Health Knowledge (MHK) and Recognition and Familiarity with Various Mental Health Conditions (RFMH) Items with Sociodemographic Variables.

V	S	B	C	EI	Bridge_EI1	Bridge_EI2
I1	0.220	0	0.001	0.170	0.089	0.137
I2	0.580	30	0.001	0.650	0.418	0.610
I3	0.450	24	0.001	0.503	0.133	0.339
I4	0.550	8	0.001	0.520	0.075	0.246
I5	0.350	28	0.001	0.324	0.131	0.273
I6	0.060	0	0.001	−0.024	−0.016	−0.030
I7	1.000	120	0.002	1.162	0.194	0.403
I8	0.620	32	0.001	0.761	0.000	0.066
I9	0.820	22	0.002	0.867	0.253	0.499
I10	1.020	130	0.002	1.141	0.314	0.545
I11	0.700	16	0.002	0.739	0.169	0.309
I12	0.600	0	0.001	0.494	0.000	0.013
Sex	0.550	0	0.000	−0.003	−0.003	−0.007
Age	0.560	32	0.001	0.475	−0.006	−0.024
Edu	0.280	0	0.001	0.219	0.219	0.404
WE	0.780	0	0.001	0.490	0.009	0.010
MHE	0.470	2	0.001	0.203	0.203	0.270
CF	0.180	0	0.001	0.104	0.104	0.207

Note: V = Variables, S = Strength, B = Betweenness, C = Closeness, EI = Expected Influence, Bridge_EI1 = Bridge Expected Influence 1° Step Bridge_EI2 = Bridge Expected Influence 2° Step, I1–I6 = Items Assessing Mental Health Knowledge, I7–I12 = Recognition and Familiarity with Various Mental Health Conditions, Edu = Education, MHE = Mental Health Education, CF = Contact Frequency with a Person Diagnosed with Mental Disorder, WE = Work Experience.

## Data Availability

The data that support the findings of this study are available upon request from the corresponding author.

## Abbreviations

The following abbreviations are used in this manuscript:

PHC	Primary Health Care
MAKS	Mental Health Knowledge Schedule
